# Sleep quality, internet addiction and depressive symptoms among undergraduate students in Nepal

**DOI:** 10.1186/s12888-017-1275-5

**Published:** 2017-03-21

**Authors:** Parash Mani Bhandari, Dipika Neupane, Shristi Rijal, Kiran Thapa, Shiva Raj Mishra, Amod Kumar Poudyal

**Affiliations:** 1 Maharajgunj Medical Campus, Institute of Medicine, Tribhuvan University, Kathmandu, Nepal; 2 University of Western Australia, School of Population Health, Crawley, WA Australia; 3 Nepal Development Society, Bharatpur-10, Nepal

**Keywords:** Undergraduates, Depression, Internet use, Insomnia, Nepal

## Abstract

**Background:**

Evidence on the burden of depression, internet addiction and poor sleep quality in undergraduate students from Nepal is virtually non-existent. While the interaction between sleep quality, internet addiction and depressive symptoms is frequently assessed in studies, it is not well explored if sleep quality or internet addiction statistically mediates the association between the other two variables.

**Methods:**

We enrolled 984 students from 27 undergraduate campuses of Chitwan and Kathmandu, Nepal. We assessed sleep quality, internet addiction and depressive symptoms in these students using Pittsburgh Sleep Quality Index, Young’s Internet Addiction Test and Patient Health Questionnaire-9 respectively. We included responses from 937 students in the data analysis after removing questionnaires with five percent or more fields missing. Via bootstrap approach, we assessed the mediating role of internet addiction in the association between sleep quality and depressive symptoms, and that of sleep quality in the association between internet addiction and depressive symptoms.

**Results:**

Overall, 35.4%, 35.4% and 21.2% of students scored above validated cutoff scores for poor sleep quality, internet addiction and depression respectively. Poorer sleep quality was associated with having lower age, not being alcohol user, being a Hindu, being sexually active and having failed in previous year’s board examination. Higher internet addiction was associated with having lower age, being sexually inactive and having failed in previous year’s board examination. Depressive symptoms were higher for students having higher age, being sexually inactive, having failed in previous year’s board examination and lower years of study. Internet addiction statistically mediated 16.5% of the indirect effect of sleep quality on depressive symptoms. Sleep quality, on the other hand, statistically mediated 30.9% of the indirect effect of internet addiction on depressive symptoms.

**Conclusions:**

In the current study, a great proportion of students met criteria for poor sleep quality, internet addiction and depression. Internet addiction and sleep quality both mediated a significant proportion of the indirect effect on depressive symptoms. However, the cross-sectional nature of this study limits causal interpretation of the findings. Future longitudinal study, where the measurement of internet addiction or sleep quality precedes that of depressive symptoms, are necessary to build upon our understanding of the development of depressive symptoms in students.

## Background

Prevalence rate of poor sleep quality [1], internet addiction [2] and depressive symptoms [3] is high among undergraduate students worldwide. In Nepal, mobile penetration rate has crossed cent percent while the internet penetration rate alone has reached 54.34% [4]. However, internet addiction in undergraduate students from Nepal is not studied so far but it can be estimated to be highly prevalent since majority of the internet users are aged 18–24 years [5], typical age-group of undergraduate students. Likewise, the proportion of Nepalese aged 18–25 years experiencing depression is 5.61% [6]; however, depression in Nepalese undergraduate students is not explored till date. Similarly, sleep quality in Nepalese undergraduates has not been studied, but 55.8% of undergraduates were found to have poor sleep quality in a similar low-resource setting [7]. Overall, the evidence on burden of sleep quality, internet addiction and depression in Nepalese undergraduate students is completely lacking.

An earlier study showed that students with sleep problems spend more time watching television and surfing the social networking websites [8]. These students — who spend greater time in internet — are more likely to develop depressive symptoms [9]. On the other hand, internet addict students have higher chance of experiencing sleep problems [10]. These students — with sleep problems — are more likely to develop depressive symptoms [11]. These evidences indicate that the interplay between sleep quality, internet addiction and depressive symptoms is somewhat complex and can be theorized via two distinct pathways. In the first postulated pathway, internet addiction mediates the relation between sleep quality and depressive symptoms. In the second postulated pathway, sleep quality mediates the relation between internet addiction and depressive symptoms. Exploring these pathways and determining which pathway is more plausible will enrich our understanding of the development of depressive symptoms in students. More importantly, it will help to understand predictors of depressive symptoms and aid in devising effective interventions to stem depression in students.

Interestingly, only a single study has assessed these two proposed mediation pathways till date [12]. In their study, Cheung and Wong observed that sleep quality accounted for 5% of the association between internet use and depressive symptoms while internet use accounted for 12% of the association between sleep quality and depression; both mediation pathways being statistically significant. The study used causal steps approach to arrive at the conclusion that both internet addiction and sleep quality significantly mediates depression. Causal steps approach of testing mediation, however, is reported to have several limitations. It can lead researchers to a fallacious conclusion with its unnecessarily stringent series of hypothesis testing and the absence of a formal statistic to quantify the indirect effect [13]. So, testing if their findings could be replicated in another population using bootstrap approach will be helpful in exploring the complex interplay between sleep quality, internet addiction and depressive symptoms.

Considering the aforementioned gaps, in this study we focused on answering two research questions. First, we assessed the proportion of Nepalese undergraduate students meeting criteria for poor sleep quality, depression and internet addiction, and explored their correlates. Second, we investigated the proposed mediation models using bootstrap approach: if internet addiction statistically mediated the association between sleep quality and depressive symptoms, and also if sleep quality statistically mediated the association between internet addiction and depressive symptoms.

## Methods

### Study setting

This cross-sectional study was conducted in 27 undergraduate campuses in Kathmandu and Chitwan districts of Nepal. These are two of the districts having very high number of campuses per district according to the University Grants Commission Report [14]. Kathmandu district is the national capital and the largest metropolitan, and Chitwan is another district located 146 km west of Kathmandu in the southern plains.

### Student recruitment

In our study, we enrolled 984 undergraduate students considering a power 0.85, α 0.05, effect size 0.01 and non-response rate 8%. First, we selected Kathmandu and Chitwan districts purposively. Second, we randomly selected 27 campuses in proportion to the total number of campus in each district: 7 from Chitwan and 20 from Kathmandu using the sampling frame obtained from the University Grants Commission. Third, from the class-roster available on the day of data collection at each selected campus, one class was selected randomly. Fourth, we invited all students in the selected classroom to participate in this study and collected data using self-administered questionnaires. Data collection was carried out in between September and November 2015.

### Measures

The questionnaire, originally developed in English, contained questions on students' characteristics, sleep quality, internet addiction and depressive symptoms. The questionnaire was translated to Nepali and back-translated into English independently to make sure the original meaning remains unchanged. The questionnaire was pretested in 20 undergraduate students of public health, who were not the part of final survey. The internal consistency of final translated questionnaire, in this study population, was assessed with Cronbach’s α.

#### Students’ characteristics

Information on age, sex, caste, religion, marital status, family size, employment status, tobacco use, alcohol use, sexual activity, academic performance in previous year, study year, campus type and study shift was obtained from the students. These variables, selected after literature review [1, 3, 15], are not an exhaustive collection of correlates but include major correlates of sleep quality, internet addiction and depressive symptoms among students. We categorized castes into two groups ‘Brahmin/Chhetri’ and ‘Others’. This grouping was based on the differential social rank allocated traditionally to different castes based on their dominancy in Nepalese societies [16]. We grouped students based on their use of tobacco and alcoholic beverages in past 30 days. Students who had consumed a tobacco product at least once (either smoking or chewing) were defined as tobacco users. Similarly, alcohol users were defined as students who had consumed any alcoholic beverage at least once. Students’ sexual activity was recorded as ‘sexually active’ and ‘sexually inactive’. Students who had at least one event of penetrative sexual activity within the previous 30 days of data collection were categorized as sexually active. To assess academic performance of the students, outcome of board examination in the previous year was recorded: those who have succeeded were grouped as ‘Passed’ and others were grouped as ‘Failed’.

#### Sleep quality

Students’ sleep quality was assessed using Pittsburgh Sleep Quality Index (PSQI). PSQI is a 19-item tool that evaluates sleep quality over a period of one month. PSQI score can range from 0 to 21, in which a greater score suggests poor sleep quality. We used PSQI global score greater than 5, which had sensitivity of 89.6% and specificity of 86.5% [17], to determine if the student met criteria for poor sleep quality. On the other hand, PSQI score in continuous scale was used for the mediation analysis. The internal consistency of PSQI in the current study was low (Cronbach’s α = 0.610).

#### Internet addiction

Internet addiction of the undergraduate students was assessed using Young’s 20-item Internet Addiction Test (IAT). Score for individual item of IAT can range from 1 (rarely) to 5 (always). Composite score for IAT can range from 20 to 100 where a greater score is indicative of greater internet addiction. IAT score greater than or equal to 40, as suggested previously [18], was used to determine if the student met the criteria for internet addiction. In the mediational model, IAT score in continuous scale was used instead. Internal consistency of IAT in this study population was excellent (Cronbach’s α = 0.834).

#### Depressive symptoms

Depressive symptoms were assessed by nine-item scale, Patient Health Questionnaire-9 (PHQ-9). Score for each item of PHQ-9 ranges from 0 (not at all) to 3 (almost every day) based on the frequency of symptoms experienced. The total score ranges from 0 to 27, greater score reflecting more frequent and higher number of depressive symptoms. PHQ-9 score of greater than or equal to 10, that had sensitivity of 88% and specificity of 88% [19], was used to determine if the student met criteria for depression. For the mediation analysis, we used the PHQ-9 score in continuous scale instead. The Cronbach’s α for PHQ-9 in this study population was 0.809.

### Statistical analysis

We collected data from 984 undergraduate students, removed 47 questionnaires with 5% or more fields (three fields or more) missing, yielding only 937 questionnaires for statistical analysis. Missing fields, if any, for these 937 questionnaires were replaced by the median value for that particular variable.

To establish correlates, we entered all the variables into three separate multivariable linear regression models; one each for sleep quality, internet addiction and depressive symptoms. Bootstrap models with 5000 replications were employed to calculate stable estimates of correlates.

For the best test of mediation effect, non- parametric bootstrap approach, which is not based on the assumption of normal distribution [20], was used. Bootstrap approach provides an estimate of indirect effect, tests whether the effect is statistically significant and also determines the confidence interval for the point estimate, which tends to supplement the limitations of causal steps approach. This approach suggests that the mediation effect is significant if the bias accelerated and corrected (BCa) confidence intervals do not include a zero. Additionally, it is advised to consider potential mediator-outcome confounders while running mediational models to account for potential interaction of confounding variables [21]. So, we first ran an unadjusted mediational model with the exposure, mediator and outcome variable. Second, we re-ran the mediational model adjusting for socio-demographics. Third, we added behavioral variables as covariates into the mediational model. Finally, we ran the mediational model adjusting for all socio-demographics, behavioral variables and educational variables. We derived coefficients of effects and their BCa 95% confidence intervals from 5000 random bootstrap samples. All the analyses were carried out in IBM SPSS Statistics 20. PROCESS macro was used for the mediational analyses.

## Ethics

The study protocol was approved by Nepal Health Research Council (Ref No. 196, 2015). Study objectives were explained to students and their written informed consent was obtained prior to the data collection. None of the students denied participating after being approached for consent. After data collection, a ten minutes informative session on symptoms of poor sleep quality, internet addiction and depression was conducted. Students were suggested to visit the nearby health centre for further clinical consultation if they had many of those symptoms.

## Results

This study comprised 937 undergraduate students (mean age: 21.01 ± 2.18 years, 54.6% female). Majority of the students were Hindu (92.2%) and Brahmin/Chhetri (70.7%). Most of the students studied in private campus (54.5%), attended morning shift of class (59.3%), were unemployed (85.2%) and had passed board examination of previous year (90.7%). Percentage of students using tobacco was 9.1% and that who consumed alcohol was 19.1%. Similarly, 20.9% of the students were sexually active.

### Prevalence of poor sleep quality, internet addiction and depressive symptoms, and their correlates

For PSQI, the mean score was 4.91 (SD: 2.40) and the students’ minimum score was 0 while the maximum score was 15. The mean score for IAT was 37.12 (SD: 8.48) with the students’ score ranging from minimum of 20 and maximum of 89. The mean score for PHQ-9 was 6.80 (SD: 4.22) with the students’ minimum score of 0 and maximum score of 25. The proportion of students meeting criteria for both poor sleep quality (PSQI > 5) and internet addiction (IAT ≥ 40) were 35.4%. Similarly, 21.2% of the students met criteria for depression (PHQ-9 ≥ 10). Students having at least one of the three conditions (poor sleep quality, internet addiction or depression) constituted 55.6% and those with all of the three conditions were 10.1%.

The results from multivariable linear regression models of the relationship of students’ characteristics with sleep quality, internet addiction and depressive symptoms are tabled in Table 1. Overall, students' higher age was associated with better sleep quality (b = −0.089; BCa 95% CI = −0.164, −0.008; *p* < 0.05). Although no significant relation between students' tobacco use and sleep quality was found, students who were alcohol users had better sleep quality (b = −0.777; BCa 95% CI = −1.272, −0.301; *p* < 0.05). In comparison to Hindu group, Non-Hindu students had better sleep quality (b = −0.499; BCa 95% CI = −0.997, −0.002; *p* < 0.05). Student’s sexual activity was observed to be associated with sleep quality; in comparison to sexually inactive students, sexually active students had poorer sleep quality (b = 0.514; BCa 95% CI = 0.097, 0.940; *p* < 0.05). Students who reported to having passed in their previous year’s board examination had better sleep quality (b = −1.026; BCa 95% CI = −1.627, −0.414; *p* < 0.05).

**Table 1:** Multivariable linear regression models of students’ characteristics with sleep quality, internet addiction and depressive symptoms

Characteristics	Sleep quality		Internet addiction		Depressive symptoms	
	b	BCa 95% CI	b	BCa 95% CI	b	BCa 95% CI
Age	**−0.089**	−0.164, −0.008	**−0.476**	−0.773, −0.183	−**0.145**	−0.275, −0.024
Sex (reference: male)	−0.191	−0.537, 0.148	**−2.362**	−3.530, −1.267	0.471	−0.139, 1.099
Caste (reference: Brahmin/Chhetri)	0.253	−0.077, 0.609	−0.017	−1.337, 1.339	0.347	−0.255, 0.949
Religion (reference: Hindu)	**−0.499**	−0.997, −0.002	−0.205	−2.448, 2.096	−0.186	−1.127, 0.770
Marital status (reference: married)	−0.205	−1.315, 0.843	0.159	−4.123, 4.173	1.143	−0.338, 2.561
Family size	0.017	−0.057, 0.092	−0.072	−0.297, 0.167	−0.102	−0.213, 0.017
Employment status (reference: employed)	−0.315	−0.763, 0.111	0.216	−1.240, 1.618	−0.631	−1.476, 0.210
Tobacco use (reference: users)	0.362	−0.387, 1.100	0.618	−1.830, 3.192	−0.546	−1.701, 0.607
Alcohol use (reference: users)	**−0.777**	−1.272, −0.301	−1.050	−2.811, 0.688	−0.478	−1.305, 0.328
Sexual activity (reference: sexually inactive)	**0.514**	0.097, 0.940	**2.099**	0.510, 3.700	**1.187**	0.384, 1.994
Academic performance in previous year (reference: failed)	**−1.026**	−1.627, −0.414	**−2.431**	−4.213, −0.751	**−2.041**	−3.125, −0.963
Study year	−0.123	−0.277, 0.031	0.053	−0.479, 0.584	−**0.336**	−0.586, −0.078
Campus type (reference: public)	−0.039	−0.355, 0.282	**1.342**	0.192, 2.459	−0.183	−0.739, 0.359
Study shift (reference: morning)	−0.189	−0.524, 0.143	**1.864**	0.602, 3.072	0.255	−0.281, 0.799

Statistically significant associations are highlighted in bold

b: unstandardized coefficient; BCa: Bias corrected and accelerated: 5000 bootstrap samples

Both having higher age (b = −0.476; BCa 95% CI = −0.773, −0.183; *p* < 0.05) and being female (b = −2.362; BCa 95% CI = −3.530, −1.267; *p* < 0.05) were associated with lower internet addiction. Sexually active students, in comparison to those sexually inactive, had higher internet addiction (b = 2.099; BCa 95% CI = 0.510, 3.700; *p* < 0.05). Having passed the previous year’s board examination was associated with lower internet addiction (b = −2.431; BCa 95% CI = −4.213, −0.751; *p* < 0.05). Similarly, students who studied in a private campus (b = 1.342; BCa 95% CI = 0.192, 2.459; *p* < 0.05) and those who studied in a day shift (b = 1.864; BCa 95% CI = 0.602, 3.072; *p* < 0.05) had higher rates of internet addiction.

Higher age was associated with lower depressive symptoms in students (b = −0.145; BCa 95% CI = −0.275, −0.024; *p* < 0.05). Higher rate of depressive symptoms was observed in sexually active students (b = 1.187; BCa 95% CI = 0.384, 1.994; *p* < 0.05). Students who had passed their previous board examination had lower depressive symptoms than their counterparts who had failed (b = −2.041; BCa 95% CI = −3.125, −0.963; *p* < 0.05). Interestingly, with greater number of years of undergraduate study, depressive symptoms became lower (b = −0.336; BCa 95% CI = −0.586, −0.078; *p* < 0.05).

### Mediation of association between sleep quality and depressive symptoms by internet addiction

Table 2 presents the findings from mediational analysis with internet addiction as the mediator. The unadjusted model, Model 1 suggested that 16.9% of the indirect effect of sleep quality on depressive symptoms was statistically mediated by internet addiction in students. In Model 2, after entering socio-demographics as covariates into the mediational model, the proportion of indirect effect mediated via internet addiction dropped down to 16.8%. Further, when we added behavior-related variables as covariates in Model 3, the proportion of indirect effect mediated by internet addiction was further reduced to 16.4%. In the final model, Model 4, we added education-related variables to existing covariates. Internet addiction mediated 16.5% of the indirect effect of sleep quality on depressive symptoms in this final model. In all of the four mediational models, mediation of association between sleep quality and depressive symptoms by internet addiction was statistically significant.

**Table 2:** Effect estimates of effects of sleep quality on depressive symptoms in undergraduate students mediated via internet addiction

	Model 1		Model 2		Model 3		Model 4	
	b (SE)	BCa 95% CI	b (SE)	BCa 95% CI	b (SE)	BCa 95% CI	b (SE)	BCa 95% CI
Indirect effect	0.146 (0.024)	0.103, 0.197	0.145 (0.024)	0.103, 0.198	0.140 (0.024)	0.098, 0.192	0.138 (0.025)	0.096, 0.192
Direct effect	0.717 (0.051)	0.617, 0.817	0.720 (0.051)	0.620, 0.820	0.713 (0.051)	0.613, 0.814	0.697 (0.052)	0.596, 0.798
Total effect	0.863 (0.051)	0.764, 0.962	0.865 (0.051)	0.765, 0.965	0.853 (0.051)	0.753, 0.954	0.835 (0.051)	0.735, 0.936
Proportion of total effect mediated	0.169		0.168		0.164		0.165	
Ratio of indirect to direct effect	0.204		0.201		0.196		0.198	

Model 1: unadjusted mediational model

Model 2: adjusted for sociodemographics (age, sex, caste, religion, marital status, family size, employment status)

Model 3: adjusted for sociodemographics + behavioral variables (tobacco use, alcohol use, sexual activity)

Model 4: adjusted for sociodemographics + behavioral variables + educational variables (class hours, academic year, campus type, academic performance in previous year)

b: unstandardized coefficient; BCa, Bias corrected and accelerated: 5000 bootstrap samples

### Mediation of association between internet addiction and depressive symptoms by sleep quality

Findings from the mediational analysis with internet addiction as the mediator is presented in Table 3. In the unadjusted model i.e. Model 1, the proportion of indirect effect of internet addiction on depressive symptoms mediated by sleep quality was 33.0%. This proportion dropped down to 32.0% in Model 2 when sociodemographics were entered into the model. After adding behavior-related variables into the mediation model, the proportion of indirect effect on depressive symptoms mediated by sleep quality dropped down to 31.1%. Finally, after adding education-related variables to the model, sleep quality mediated 30.9% of the indirect effect of internet addiction on depressive symptoms. Mediation of association between internet addiction and depressive symptoms via sleep quality was statistically significant in all the four models.

**Table 3:** Effect estimates of effects of internet addiction on depressive symptoms in undergraduate students mediated via sleep quality

	Model 1		Model 2		Model 3		Model 4	
	b (SE)	BCa 95% CI	b (SE)	BCa 95% CI	b (SE)	BCa 95% CI	b (SE)	BCa 95% CI
Indirect effect	0.064 (0.008)	0.049, 0.081	0.063 (0.008)	0.048, 0.080	0.060 (0.008)	0.046, 0.078	0.059 (0.008)	0.044, 0.076
Direct effect	0.130 (0.014)	0.101, 0.158	0.134 (0.015)	0.106, 0.163	0.133 (0.015)	0.104, 0.161	0.132 (0.015)	0.103, 0.161
Total effect	0.194 (0.015)	0.164, 0.223	0.197 (0.015)	0.167, 0.227	0.193 (0.015)	0.163, 0.223	0.191 (0.015)	0.161, 0.221
Proportion of total effect mediated	0.330		0.320		0.311		0.309	
Ratio of indirect to direct effect	0.493		0.469		0.453		0.446	

Model 1: unadjusted mediational model

Model 2: adjusted for sociodemographics (age, sex, caste, religion, marital status, family size, employment status)

Model 3: adjusted for sociodemographics + behavioral variables (tobacco use, alcohol use, sexual activity)

Model 4: adjusted for sociodemographics + behavioral variables + educational variables (class hours, academic year, campus type, academic performance in previous year)

b: unstandardized coefficient; BCa, Bias corrected and accelerated: 5000 bootstrap samples

## Discussion

We found that a high percentage of undergraduate students met criteria for internet addiction, poor sleep quality and depression- more than half of the students had at least one of these problems. We explored the differential effects of poor sleep quality and internet addiction on depressive symptoms. In our study, on one hand, internet addiction independently mediated 16.5% of the indirect effect of sleep quality on depressive symptoms while on the other hand, sleep quality independently mediated 30.9% of the indirect effect of internet addiction on depressive symptoms. This suggests that it is not entirely sleep quality or internet addiction per se that increases risk of depressive symptoms but a battery of predictors including internet addition and sleep quality that affects depressive symptoms. Sleep quality mediated a greater proportion of the indirect effect on depressive symptoms in comparison to the internet addiction. This finding is in contrast to what Cheung and Wong found where internet addiction accounted for larger association between sleep quality and depressive symptoms [12]. This contrasting finding is interesting to explore further in relation to the target population, and geographical and technological landscape.

Our study confirms a huge proportion of undergraduate students (one in three) meeting criteria for poor sleep quality. This can be attributed to the change in sleep pattern in the study years; physiological need of sleep does not commensurate with the sleep quality during this period [22]. Moreover, societal and academic demands perturb their sleep habit making them more prone to develop poor sleep quality [11]. Our study quantified the prevalence of internet addiction in Nepalese undergraduate students showing that a large proportion of them (35.5%) indulge into internet addiction. Younger generations, whether it may be for recreation, communication or academic purpose, are more vulnerable to internet addiction [23, 24].

Co-morbidity of poor sleep quality, depressive symptoms and other psychosomatic symptoms with internet addiction is proven with ubiquitous evidences [23, 24]. General studies suggest that older age, physical inactivity, smoking, and alcohol consumption are associated with poor sleep quality; loneliness, personality and lack of social support with internet addiction; and less social support, physical inactivity, physical and sexual abuse, and life stress with depressive symptoms. Romano and colleagues observed that brief exposure to internet for fifteen minutes was associated with depressive symptoms [25]. The factors that predict depressive symptoms among university students might be different because of their exposure to different circumstances. Our study revealed that higher age was significantly associated with poor sleep quality and depressive symptoms, while inverse relation was found between age and internet addiction. Sexual activeness and poor academic performance were associated with poor sleep quality, internet addiction and depressive symptoms. Better sleep quality found among alcohol users was an interesting result of this study. Contemporary literature suggests that sleep problems are frequent among alcohol users [26]. So, this observation needs to be specifically studied in relation to operationalization and conceptualization.

We have multiple explanations for the ways internet addiction may contribute to depressive symptoms. Students may spend extended hours in social networking sites potentially harming their education. This explanation is supported by previous studies linking internet addiction with reduced academic performance [27]. Alternatively, internet addiction isolates students from social company and forces to seek a company in the virtual world instead. In a long run, they end up losing their real-life social connection [27], which is a crucial trigger in the development of depressive symptoms.

The mediation of the association between internet addiction and depressive symptoms also appears plausible in light of the available evidences. Students who are internet addicts might spend a considerable amount of their time surfing internet which potentially affects their sleep-wake schedule and develop sleep disturbances including insomnia [28]. This consequently leads to development of depressive symptoms as many previous studies have shown [29]. Despite assumptions, the definite causal pathway to depressive symptoms still remains hard to assess.

The findings from this study reveals more on the prevalence of psychological/behavioral co-morbidities in undergraduate students with depressive symptoms. Co-occurrence of poor sleep quality, internet addiction and depressive symptoms in undergraduate students suggests that control over internet addiction in students with poor sleep quality may be also beneficial to compensate for the higher likelihood of bearing depressive symptoms. Also, strategies to address poor sleep quality among internet addicts might be helpful to curb the pathway to depressive symptoms to some extent. Recently, a randomized controlled trial suggested that internet-based cognitive behavior therapy targeting insomnia was effective to reduce depressive symptoms [30]. Since the findings from our study suggest that internet addiction is co-prevalent in undergraduate students with depressive symptoms, it indicates the prospect of designing effective, tailored interventions to combat depressive symptoms alongside internet addiction in undergraduate students.

On one hand, poor sleep quality escalates internet addiction while on the other hand, it controls academic performance. In our study we found, academic performance in previous year was associated with sleep quality, internet addiction and depressive symptoms. So, to stem the burden of depressive symptoms among undergraduate students, educational institution can play a vital role. Adjusting class hours, considering physiological need for sleep, can be a good approach. Sensitizing students about negative effects of internet addiction and encouraging them to use it for productive purpose is deemed necessary.

Internal consistency of IAT and PHQ-9 in this study was high while that of PSQI was low (Cronbach’s α = 0.61). Omission of any PSQI component score did not result in a substantial increment of the alpha value. Globally, PSQI is reported to have good, though not ideal, Cronbach’s α [31]. However, this is not the first study to document poor Cronbach’s α of PSQI. Low Cronbach’s α of PSQI was also observed in previous studies assessing sleep quality in people with chronic fatigue syndrome [32], adults aged 18–59 years [33], and older men aged 65 years or more [34]. Contextualization of questionnaire items and validation of Nepali version of PSQI seems necessary if it is to be used to assess sleep quality in Nepali population in the future.

However, the findings of this study can be interpreted in the light of study limitations. The risk behaviors and their association being studied cross-sectionally, it is impossible to infer directionality in the relationship among internet addiction, sleep quality and depressive symptoms; whether depressive symptoms is secondary to internet addiction and sleep quality, or it predicts the other two. Alternatively, sleep problem could serve as outcome of internet addiction and depressive symptoms theoretically. These pathways have not been tested in this study considering the study objectives. Recently, Tan and colleagues explored these pathways in which they observed that internet addiction and depressive symptoms both had ‘partially mediating effect’ on sleep quality [35]. Longitudinal in-depth analysis in future studies, where the predictor and mediator are measured ahead of outcome variable of interest, will provide a more robust evidence of the pathway in development of depressive symptoms in students. Similarly, due to the use of self-administered questionnaire, the study might have suffered from social desirability bias i.e. students’ responses may have been influenced by what they consider is socially acceptable, instead of providing actual practices. Likewise, information on students’ socioeconomic status, media use, physical inactivity, anxiety, medication use was not recorded in this study. These variables are still likely to be associated with sleep quality, internet addiction and depressive symptoms. Future studies among undergraduates from Nepal should consider exploring their association with sleep quality, internet addiction and depressive symptoms, and exploring if including them as covariates makes a significant change in the mediation models tested in this study.

## Conclusions

Our study documents a high prevalence of poor sleep quality, internet addiction and depression in undergraduate students from Nepal. Furthermore, the findings suggest that the association between sleep quality and depressive symptoms is statistically mediated by internet addiction. Similarly, the association between internet addiction and depressive symptoms is also statistically mediated by sleep quality. How the mediation occurs should be explored in future researches using larger longitudinal designs with sufficient follow up across populations. The findings suggest that sleep quality and internet addiction should also be assessed during counseling sessions for depressive symptoms among undergraduate students.

## References

[1] Jiang XL, Zheng XY, Yang J, Ye CP, Chen YY, Zhang ZG, Xiao ZJ (2015). A systematic review of studies on the prevalence of insomnia in university students. Public Health.

[2] Yang CY, Sato T, Yamawaki N, Miyata M (2013). Prevalence and risk factors of problematic internet use: a cross-national comparison of Japanese and Chinese university students. Transcult Psychiatry.

[3] Ibrahim AK, Kelly SJ, Adams CE, Glazebrook C (2013). A systematic review of studies of depression prevalence in university students. J Psychiatr Res.

[4] Nepal Telecommunications Authority: MIS Report. Vol. 145. Kathmandu: Nepal Telecommunications Authority; 2016.

[5] Seybert H (2011). Internet use in households and by individuals in 2011. Eurostat statistics in focus.

[6] Risal A, Manandhar K, Linde M, Steiner TJ, Holen A (2016). Anxiety and depression in Nepal: prevalence, comorbidity and associations. BMC Psychiatry.

[7] Lemma S, Gelaye B, Berhane Y, Worku A, Williams MA (2012). Sleep quality and its psychological correlates among university students in Ethiopia: a cross-sectional study. BMC Psychiatry.

[8] Tavernier R, Willoughby T (2014). Sleep problems: predictor or outcome of media use among emerging adults at university?. J Sleep Res.

[9] Ko CH, Liu TL, Wang PW, Chen CS, Yen CF, Yen JY (2014). The exacerbation of depression, hostility, and social anxiety in the course of internet addiction among adolescents: a prospective study. Compr Psychiatry.

[10] Chen YL, Gau SS (2016). Sleep problems and internet addiction among children and adolescents: a longitudinal study. J Sleep Res.

[11] Roane BM, Taylor DJ (2008). Adolescent insomnia as a risk factor for early adult depression and substance abuse. Sleep.

[12] Cheung LM, Wong WS (2011). The effects of insomnia and internet addiction on depression in Hong Kong Chinese adolescents: an exploratory cross-sectional analysis. J Sleep Res.

[13] Hayes AF (2013). An introduction to mediation, moderation, and conditional process analysis: a regression-based approach.

[14] University Grants Commission: Annual Report. Bhaktapur: University Grants Commission; 2013.

[15] Widyanto L (2006). Griffiths M: ‘internet addiction’: a critical review. Int J Ment Health Addict.

[16] Gellner DN (2007). Caste, ethnicity and inequality in Nepal. Econ Polit Weekly.

[17] Buysse DJ, Reynolds CF, Monk TH, Berman SR, Kupfer DJ (1989). The Pittsburgh sleep quality index: a new instrument for psychiatric practice and research. Psychiatry Res.

[18] Fernandez-Villa T, Molina AJ, Garcia-Martin M, Llorca J, Delgado-Rodriguez M, Martin V (2015). Validation and psychometric analysis of the internet addiction test in Spanish among college students. BMC Public Health.

[19] Kroenke K, Spitzer RL, Williams JB (2001). The PHQ-9: validity of a brief depression severity measure. J Gen Intern Med.

[20] Preacher KJ, Hayes AF (2008). Asymptotic and resampling strategies for assessing and comparing indirect effects in multiple mediator models. Behav Res Methods.

[21] VanderWeele TJ (2016). Mediation analysis: a Practitioner's guide. Annu Rev Public Health.

[22] Dahl RE, Lewin DS (2002). Pathways to adolescent health sleep regulation and behavior. J Adolesc Health.

[23] Tsai HF, Cheng SH, Yeh TL, Shih CC, Chen KC, Yang YC, Yang YK (2009). The risk factors of internet addiction--a survey of university freshmen. Psychiatry Res.

[24] Young KS (1996). Psychology of computer use: XL. Addictive use of the internet: a case that breaks the stereotype. Psychol Rep.

[25] Romano M, Osborne LA, Truzoli R, Reed P. Differential psychological impact of internet exposure on internet addicts. PLoS One. 2013:8(2):e55162. 10.1371/journal.pone.0055162 PMC3567114 23408958

[26] Angarita GA, Emadi N, Hodges S, Morgan PT (2016). Sleep abnormalities associated with alcohol, cannabis, cocaine, and opiate use: a comprehensive review. Addict Sci Clin Pract.

[27] Tonioni F, D'Alessandris L, Lai C, Martinelli D, Corvino S, Vasale M, Fanella F, Aceto P, Bria P (2012). Internet addiction: hours spent online, behaviors and psychological symptoms. Gen Hosp Psychiatry.

[28] Lam LT (2014). Internet gaming addiction, problematic use of the internet, and sleep problems: a systematic review. Curr Psychiatry Rep.

[29] Baglioni C, Battagliese G, Feige B, Spiegelhalder K, Nissen C, Voderholzer U, Lombardo C, Riemann D (2011). Insomnia as a predictor of depression: a meta-analytic evaluation of longitudinal epidemiological studies. J Affect Disord.

[30] Christensen H, Batterham PJ, Gosling JA, Ritterband LM, Griffiths KM, Thorndike FP, Glozier N, O'Dea B, Hickie IB, Mackinnon AJ (2016). Effectiveness of an online insomnia program (SHUTi) for prevention of depressive episodes (the GoodNight study): a randomised controlled trial. Lancet Psychiatry.

[31] Mollayeva T, Thurairajah P, Burton K, Mollayeva S, Shapiro CM, Colantonio A (2016). The Pittsburgh sleep quality index as a screening tool for sleep dysfunction in clinical and non-clinical samples: a systematic review and meta-analysis. Sleep Med Rev.

[32] Mariman A, Vogelaers D, Hanoulle I, Delesie L, Tobback E, Pevernagie D (2012). Validation of the three-factor model of the PSQI in a large sample of chronic fatigue syndrome (CFS) patients. J Psychosom Res.

[33] Magee CA, Caputi P, Iverson DC, Huang X-F (2008). An investigation of the dimensionality of the Pittsburgh sleep quality index in Australian adults. Sleep Biol Rhythms.

[34] Spira AP, Beaudreau SA, Stone KL, Kezirian EJ, Lui LY, Redline S, Ancoli-Israel S, Ensrud K, Stewart A (2012). Reliability and validity of the Pittsburgh sleep quality index and the Epworth sleepiness scale in older men. J Gerontol A Biol Sci Med Sci.

[35] Tan Y, Chen Y, Lu Y, Li L. Exploring associations between problematic internet use, depressive symptoms and sleep disturbance among southern Chinese adolescents. Int J Environ Res Public Health. 2016;13(3):313. 10.3390/ijerph13030313 PMC4808976 26985900

